# Poly[*trans*-diaquabis­[μ_2_-2-(pyridin-3-yl)acetato-κ^2^ *N*:*O*]­zinc]

**DOI:** 10.1107/S1600536811038190

**Published:** 2011-09-30

**Authors:** Yue-Hua Li, Lin Du, Zong-Ze Li, Qi-Hua Zhao

**Affiliations:** a School of Chemical Science and Engineering, Yunnan University, Kunming 650091, People’s Republic of China; b School of Pharmacy, Dali University, Dali 671000, People’s Republic of China

## Abstract

In the title coordination polymer, [Zn(C_7_H_6_NO_2_)_2_(H_2_O)_2_]_*n*_, the Zn^II^ cation is located on an inversion center and is coordinated by four pyridyl­acetate anions and two water mol­ecules in a distorted ZnN_2_O_4_ octa­hedral geometry. The pyridine-N and carboxyl­ate-O atoms of the pyridyl­acetate anion connect to two Zn^II^ cations, forming a two-dimensional polymeric complex extending parallel to (212). Inter­molecular O—H⋯O and weak C—H⋯O hydrogen bonding is present in the crystal structure.

## Related literature

For related complexes with pyridyl­acetate ligands, see: Li *et al.* (2004 ▶); Du *et al.* (2006 ▶); Martin *et al.* (2007 ▶); Qin *et al.* (2007 ▶); Aakeröy *et al.* (1999 ▶); Evans & Lin (2002 ▶); Tong *et al.* (2003 ▶).
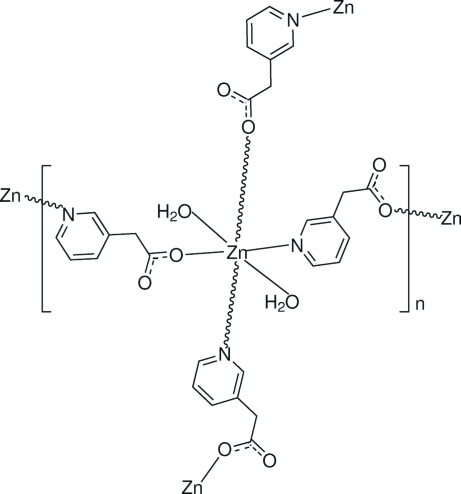


## Experimental

### 

#### Crystal data

[Zn(C_7_H_6_NO_2_)_2_(H_2_O)_2_]
*M* *_r_* = 373.66
Monoclinic, 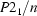

*a* = 9.175 (2) Å
*b* = 8.686 (2) Å
*c* = 9.574 (2) Å
β = 105.928 (3)°
*V* = 733.8 (3) Å^3^
*Z* = 2
Mo *K*α radiation
μ = 1.71 mm^−1^
*T* = 298 K
0.20 × 0.20 × 0.19 mm

#### Data collection

Bruker APEXII CCD area-detector diffractometer
Absorption correction: multi-scan (*SADABS*; Bruker, 2001 ▶) *T*_min_ = 0.718, *T*_max_ = 0.723
4934 measured reflections
1732 independent reflections
1178 reflections with *I* > 2σ(*I*)
*R*_int_ = 0.054

#### Refinement

*R*[*F* ^2^ > 2σ(*F* ^2^)] = 0.042
*wR*(*F* ^2^) = 0.098
*S* = 1.00
1732 reflections
112 parameters
3 restraints
H atoms treated by a mixture of independent and constrained refinement
Δρ_max_ = 0.39 e Å^−3^
Δρ_min_ = −0.38 e Å^−3^

### 

Data collection: *APEX2* (Bruker, 2007 ▶); cell refinement: *SAINT* (Bruker, 2007 ▶); data reduction: *SAINT*; program(s) used to solve structure: *SHELXTL* (Sheldrick, 2008 ▶); program(s) used to refine structure: *SHELXTL*; molecular graphics: *SHELXTL*; software used to prepare material for publication: *SHELXTL*.

## Supplementary Material

Crystal structure: contains datablock(s) I, global. DOI: 10.1107/S1600536811038190/xu5324sup1.cif

Structure factors: contains datablock(s) I. DOI: 10.1107/S1600536811038190/xu5324Isup2.hkl

Additional supplementary materials:  crystallographic information; 3D view; checkCIF report

## Figures and Tables

**Table 1:** Selected bond lengths (Å)

Zn1—N1	2.168 (3)
Zn1—O2^i^	2.091 (2)
Zn1—O3	2.125 (2)

Symmetry code: (i) 
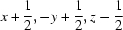
.

**Table 2:** Hydrogen-bond geometry (Å, °)

*D*—H⋯*A*	*D*—H	H⋯*A*	*D*⋯*A*	*D*—H⋯*A*
O3—H3*B*⋯O1^i^	0.81 (3)	1.99 (3)	2.739 (4)	152 (4)
O3—H3*C*⋯O1^ii^	0.82 (3)	1.97 (3)	2.764 (4)	161 (3)
C1—H1*A*⋯O1^iii^	0.93	2.54	3.443 (5)	163
C3—H3*A*⋯O1^iv^	0.93	2.50	3.366 (5)	155

Symmetry codes: (i) 
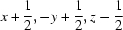
; (ii) 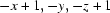; (iii) 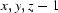; (iv) 
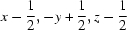
.

**Fig. 1.**
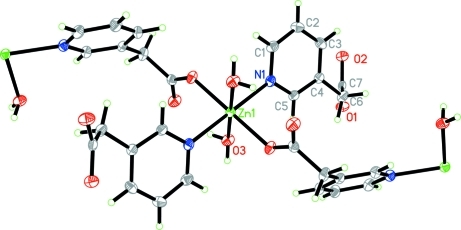
The molecular structure of the title complex with the atom-numbering diagram. Ellipsoids were drawn at the 30% probability level.

**Fig. 2.**
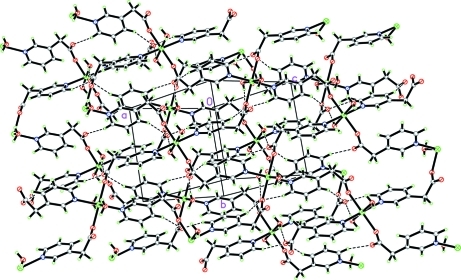
The packing diagram of (I).

## supplementary crystallographic information

### Comment

The compounds of pyridine-carboxylic acids have been extensively utilized in the preparation of metal complexes due to their versatile coordination modes. Though various metal-pyridinepolycarboxylate complexes have been reported (Evans *et al.*, 2002; Aakeröy *et al.*, 1999; Li *et al.*, 2004; Du *et al.*, 2006), 3-pyridylacetate complexes are rare. Only a few of complexes as nickel, cobalt and copper species have been combined up to now (Martin *et al.*, 2007). In this paper, we described a new two-dimensional coordination polymer, [Zn(3-pyridylacetato)_2_(H_2_O)_2_]_n_, (I). The molecular structure of the title complex is similar to those previously reported such as [*M*(4-pyridylacetato)_2_(H_2_O)_2_]_n_ (*M* = Cu, Co, Mn, Ni, Zn, Cd)(Du *et al.*, 2006; Qin *et al.*, 2007; Tong *et al.*, 2003) and [*M*(3-pyridylacetato)_2_(H_2_O)_2_]_n_ (*M* = Ni, Co, Cu) (Martin *et al.*, 2007;). Single-crystal X-ray diffraction analysis shows that the title compound is crystallized in a space group *P*2_1_/*n*. The Zn^II^ center is six-coordinated by two water molecules in the axial positions, two pyridyl nitrogen atoms and two carboxylate oxygen atoms from two 3-pyridylacetate ligands in the plane. Pyridine nitrogen atom and carboxylate oxygen atom of each 3-pyridylacetate anion are connected to one Zn^II^ ions. The coordination geometry of Zn^II^ cation can been described as a distorted octahedral geometry with Zn—N and Zn—O distance range 2.168 (2) Å and 2.091 (3)—2.125 (3) Å, respectively (Fig. 1, Table 1). Four 3-pyridylacetate anionic ligands and four Zn^II^ ions are combined to a tetragon, which is of a side length of 8.653 Å and a diagonal measurement of 14.969*8.686 Å based on the Zn—Zn distances. The tetragon is further extended into a two-dimensional framework structure parallel to (212) with arhombic grid through sharing Zn^II^ ions, 3-pyridylacetate anionic ligands. Adjacent two-dimensional layers are connected by the intermolecular O—H···O and weak C—H···O hydrogen-bonding contacts, forming a three-dimensional framework structure with oxygen as a trifurcated acceptor atom (Fig. 2)

### Experimental

A mixture of Zn(COO)_2_.H_2_O (0.1 mmol), 3-pyridyl acetic acid (0.1 mmol), DMF (5.0 ml) and methanol (10.0 ml) was stirred for 30 min and and the crude product was isolated by filtration. The filtrate was purified by recrystallization from anhydrous methanol and DMF to give (I) as colorless block crystals in 60% yield. An solution of (I) was stood at room temperature, and upon slowly evaporating methanol and DMF from the solution, colorless block crystals suitable for X-ray diffraction analysis were isolated in room temperature three week later.

### Refinement

Water H atoms were located in a difference Fourier map and positional parameters were refined, U_iso_(H) = 1.2U_eq_(O). Other H atoms were generated geometrically and were included in therefinement in the riding model approximation with C—H = 0.93–0.97 Å, U_iso_= 1.2U_eq_(C).

### Crystal data

**Table:**

[Zn(C_7_H_6_NO_2_)_2_(H_2_O)_2_]	*F*(000) = 384
*M**_r_* = 373.66	*D*_x_ = 1.691 Mg m^−^^3^
Monoclinic, *P*2_1_/*n*	Mo *K*α radiation, λ = 0.71073 Å
Hall symbol: -P 2yn	Cell parameters from 4934 reflections
*a* = 9.175 (2) Å	θ = 3.2–28.2°
*b* = 8.686 (2) Å	µ = 1.71 mm^−^^1^
*c* = 9.574 (2) Å	*T* = 298 K
β = 105.928 (3)°	Block, colorless
*V* = 733.8 (3) Å^3^	0.20 × 0.20 × 0.19 mm
*Z* = 2	

### Data collection

**Table:**

Bruker APEXII CCD area-detector diffractometer	1732 independent reflections
Radiation source: fine-focus sealed tube	1178 reflections with *I* > 2σ(*I*)
graphite	*R*_int_ = 0.054
φ and ω scans	θ_max_ = 28.2°, θ_min_ = 3.2°
Absorption correction: multi-scan (*SADABS*; Bruker, 2001)	*h* = −12→11
*T*_min_ = 0.718, *T*_max_ = 0.723	*k* = −11→11
4934 measured reflections	*l* = −9→12

### Refinement

**Table:**

Refinement on *F*^2^	Primary atom site location: structure-invariant direct methods
Least-squares matrix: full	Secondary atom site location: difference Fourier map
*R*[*F*^2^ > 2σ(*F*^2^)] = 0.042	Hydrogen site location: inferred from neighbouring sites
*wR*(*F*^2^) = 0.098	H atoms treated by a mixture of independent and constrained refinement
*S* = 1.00	*w* = 1/[σ^2^(*F*_o_^2^) + (0.035*P*)^2^ + 0.2786*P*] where *P* = (*F*_o_^2^ + 2*F*_c_^2^)/3
1732 reflections	(Δ/σ)_max_ < 0.001
112 parameters	Δρ_max_ = 0.39 e Å^−^^3^
3 restraints	Δρ_min_ = −0.38 e Å^−^^3^

### Special details

**Table:**

Geometry. All e.s.d.'s (except the e.s.d. in the dihedral angle between two l.s. planes) are estimated using the full covariance matrix. The cell e.s.d.'s are taken into account individually in the estimation of e.s.d.'s in distances, angles and torsion angles; correlations between e.s.d.'s in cell parameters are only used when they are defined by crystal symmetry. An approximate (isotropic) treatment of cell e.s.d.'s is used for estimating e.s.d.'s involving l.s. planes.
Refinement. Refinement of *F*^2^ against ALL reflections. The weighted *R*-factor *wR* and goodness of fit *S* are based on *F*^2^, conventional *R*-factors *R* are based on *F*, with *F* set to zero for negative *F*^2^. The threshold expression of *F*^2^ > σ(*F*^2^) is used only for calculating *R*-factors(gt) *etc*. and is not relevant to the choice of reflections for refinement. *R*-factors based on *F*^2^ are statistically about twice as large as those based on *F*, and *R*- factors based on ALL data will be even larger.

### Fractional atomic coordinates and isotropic or equivalent isotropic displacement parameters (Å^2^)

**Table:**

	*x*	*y*	*z*	*U*_iso_*/*U*_eq_	
Zn1	0.5000	0.0000	0.0000	0.02624 (18)	
O1	0.2006 (3)	0.1719 (3)	0.6106 (3)	0.0400 (6)	
O2	0.0333 (3)	0.2812 (3)	0.4230 (2)	0.0331 (6)	
O3	0.6282 (3)	0.0534 (3)	0.2153 (3)	0.0359 (6)	
H3C	0.694 (3)	0.002 (3)	0.272 (3)	0.043*	
H3B	0.676 (4)	0.130 (3)	0.207 (4)	0.043*	
N1	0.3007 (3)	0.0777 (3)	0.0596 (3)	0.0293 (6)	
C1	0.1913 (4)	0.1572 (4)	−0.0326 (4)	0.0346 (8)	
H1A	0.2032	0.1814	−0.1235	0.042*	
C2	0.0611 (4)	0.2054 (4)	0.0003 (4)	0.0372 (9)	
H2A	−0.0123	0.2612	−0.0670	0.045*	
C3	0.0414 (4)	0.1699 (4)	0.1341 (4)	0.0346 (8)	
H3A	−0.0460	0.2004	0.1578	0.042*	
C4	0.1537 (4)	0.0881 (4)	0.2333 (3)	0.0267 (7)	
C5	0.2802 (4)	0.0449 (4)	0.1900 (4)	0.0295 (8)	
H5A	0.3557	−0.0104	0.2555	0.035*	
C6	0.1407 (4)	0.0437 (4)	0.3815 (4)	0.0341 (9)	
H6A	0.2296	−0.0158	0.4302	0.041*	
H6B	0.0532	−0.0229	0.3692	0.041*	
C7	0.1255 (4)	0.1768 (4)	0.4799 (4)	0.0278 (7)	

### Atomic displacement parameters (Å^2^)

**Table:**

	*U*^11^	*U*^22^	*U*^33^	*U*^12^	*U*^13^	*U*^23^
Zn1	0.0299 (3)	0.0285 (3)	0.0225 (3)	−0.0017 (3)	0.0111 (2)	−0.0004 (2)
O1	0.0477 (16)	0.0369 (14)	0.0309 (14)	0.0064 (12)	0.0033 (12)	0.0006 (11)
O2	0.0417 (15)	0.0324 (13)	0.0259 (12)	0.0088 (11)	0.0106 (11)	−0.0015 (10)
O3	0.0426 (16)	0.0340 (14)	0.0279 (14)	−0.0022 (12)	0.0044 (11)	−0.0018 (11)
N1	0.0336 (17)	0.0316 (16)	0.0256 (15)	−0.0012 (13)	0.0130 (12)	0.0007 (12)
C1	0.045 (2)	0.034 (2)	0.0261 (18)	0.0034 (17)	0.0126 (16)	0.0024 (15)
C2	0.037 (2)	0.039 (2)	0.034 (2)	0.0102 (17)	0.0060 (16)	0.0004 (16)
C3	0.029 (2)	0.037 (2)	0.039 (2)	0.0047 (16)	0.0121 (16)	−0.0065 (17)
C4	0.033 (2)	0.0232 (18)	0.0263 (17)	0.0001 (14)	0.0123 (15)	−0.0021 (14)
C5	0.034 (2)	0.0275 (18)	0.0286 (18)	0.0025 (14)	0.0116 (15)	0.0035 (14)
C6	0.048 (2)	0.0259 (18)	0.035 (2)	0.0059 (16)	0.0234 (17)	0.0035 (14)
C7	0.0286 (19)	0.0282 (18)	0.0314 (19)	−0.0034 (15)	0.0160 (15)	0.0037 (15)

### Geometric parameters (Å, °)

**Table:**

Zn1—N1	2.168 (3)	C1—C2	1.382 (5)
Zn1—N1^i^	2.168 (3)	C1—H1A	0.9300
Zn1—O2^ii^	2.091 (2)	C2—C3	1.377 (5)
Zn1—O2^iii^	2.091 (2)	C2—H2A	0.9300
Zn1—O3	2.125 (2)	C3—C4	1.390 (5)
O1—C7	1.252 (4)	C3—H3A	0.9300
O2—C7	1.258 (4)	C4—C5	1.387 (4)
O2—Zn1^iv^	2.091 (2)	C4—C6	1.507 (4)
O3—H3C	0.825 (18)	C5—H5A	0.9300
O3—H3B	0.812 (17)	C6—C7	1.522 (4)
N1—C1	1.333 (4)	C6—H6A	0.9700
N1—C5	1.344 (4)	C6—H6B	0.9700
			
O2^ii^—Zn1—O2^iii^	180.00 (12)	C1—C2—H2A	120.4
O2^ii^—Zn1—O3^i^	87.23 (9)	C2—C3—C4	119.2 (3)
O2^iii^—Zn1—O3^i^	92.77 (9)	C2—C3—H3A	120.4
O2^ii^—Zn1—N1^i^	88.57 (10)	C4—C3—H3A	120.4
O2^iii^—Zn1—N1^i^	91.43 (10)	C5—C4—C3	117.3 (3)
O3^i^—Zn1—N1^i^	87.67 (10)	C5—C4—C6	120.1 (3)
O2^ii^—Zn1—N1	91.43 (10)	C3—C4—C6	122.6 (3)
O2^iii^—Zn1—N1	88.57 (10)	N1—C5—C4	124.2 (3)
O3^i^—Zn1—N1	92.33 (10)	N1—C5—H5A	117.9
N1^i^—Zn1—N1	180.00 (12)	C4—C5—H5A	117.9
C7—O2—Zn1^iv^	130.4 (2)	C4—C6—C7	115.7 (3)
H3C—O3—H3B	101 (2)	C4—C6—H6A	108.4
C1—N1—C5	117.0 (3)	C7—C6—H6A	108.4
C1—N1—Zn1	121.5 (2)	C4—C6—H6B	108.4
C5—N1—Zn1	121.5 (2)	C7—C6—H6B	108.4
N1—C1—C2	123.1 (3)	H6A—C6—H6B	107.4
N1—C1—H1A	118.4	O1—C7—O2	125.3 (3)
C2—C1—H1A	118.4	O1—C7—C6	118.3 (3)
C3—C2—C1	119.1 (3)	O2—C7—C6	116.4 (3)
C3—C2—H2A	120.4		

Symmetry codes: (i) −*x*+1, −*y*, −*z*; (ii) *x*+1/2, −*y*+1/2, *z*−1/2; (iii) −*x*+1/2, *y*−1/2, −*z*+1/2; (iv) −*x*+1/2, *y*+1/2, −*z*+1/2.

### Hydrogen-bond geometry (Å, °)

**Table:**

*D*—H···*A*	*D*—H	H···*A*	*D*···*A*	*D*—H···*A*
O3—H3B···O1^ii^	0.81 (3)	1.99 (3)	2.739 (4)	152 (4)
O3—H3C···O1^v^	0.82 (3)	1.97 (3)	2.764 (4)	161 (3)
C1—H1A···O1^vi^	0.93	2.54	3.443 (5)	163
C3—H3A···O1^vii^	0.93	2.50	3.366 (5)	155

Symmetry codes: (ii) *x*+1/2, −*y*+1/2, *z*−1/2; (v) −*x*+1, −*y*, −*z*+1; (vi) *x*, *y*, *z*−1; (vii) *x*−1/2, −*y*+1/2, *z*−1/2.
